# Progress Toward Global Eradication of Dracunculiasis — January 2012–June 2013

**Published:** 2013-10-25

**Authors:** Donald R. Hopkins, Ernesto Ruiz-Tiben, Mark L. Eberhard, Sharon L. Roy

**Affiliations:** The Carter Center, Atlanta, Georgia; Div of Parasitic Diseases and Malaria, Center for Global Health; Div of Foodborne, Waterborne, and Environmental Diseases, National Center for Emerging and Zoonotic Infectious Diseases; World Health Organization Collaborating Center for Research, Training, and Eradication of Dracunculiasis; CDC

Dracunculiasis (Guinea worm disease) is caused by *Dracunculus medinensis*, a parasitic worm. Approximately 1 year after infection from contaminated drinking water, the worm emerges through the skin of the infected person, usually on the lower limb. Pain and secondary bacterial infection can cause temporary or permanent disability that disrupts work and schooling. In 1986, the World Health Assembly (WHA) called for dracunculiasis elimination (1), and the global Guinea Worm Eradication Program, supported by The Carter Center, World Health Organization (WHO), United Nations Children’s Fund (UNICEF), CDC, and other partners, began assisting ministries of health of dracunculiasis-endemic countries in meeting this goal. At that time, an estimated 3.5 million cases occurred each year in 20 countries in Africa and Asia (1,2). This report updates published (3–5) and unpublished surveillance data reported by ministries of health and describes progress toward dracunculiasis eradication. A total of 542 cases were reported in 2012, compared with 1,058 in 2011. The disease remains endemic in four countries in 2013, but the overall rate of reduction in cases has accelerated compared with the first 6 months of 2012. In the month of January 2013, no cases were reported worldwide for the first time since the eradication program began in 1986. Failures in surveillance and containment, lack of clean drinking water, insecurity in Mali and parts of South Sudan, and an unusual epidemiologic pattern in Chad are the main remaining challenges to dracunculiasis eradication.

Because the lifecycle of *D. medinensis* is complex, its transmission can be interrupted using several strategies (4). Dracunculiasis can be prevented by 1) educating residents in dracunculiasis-endemic communities, and particularly persons from whom worms are emerging, to avoid immersing affected body parts in sources of drinking water; 2) filtering potentially contaminated drinking water through a cloth filter; 3) treating potentially contaminated surface water with the insecticide temephos (Abate); and 4) providing safe drinking water from bore-hole or hand-dug wells (6). Containment of transmission,* achieved through 1) voluntary isolation of each patient to prevent contamination of drinking water sources, 2) provision of first aid, 3) manual extraction of the worm, and 4) application of occlusive bandages, complements the four main interventions.

Countries enter the WHO precertification stage of eradication after completing 1 full calendar year without reporting any indigenous cases (i.e., one incubation period for *D. medinensis*). A case of dracunculiasis is defined as infection occurring in a person exhibiting a skin lesion or lesions with emergence of one or more Guinea worms. Each infection is counted as a case only once during a calendar year. An imported case is an infection acquired in a place (another country or village within the same country) other than the community where it is detected and reported. Six countries where transmission of dracunculiasis was previously endemic (Cote d’Ivoire, Ghana, Kenya, Niger, Nigeria, and Sudan) are in the precertification stage of eradication.

In each country affected by dracunculiasis, a national eradication program receives monthly reports of cases from each village that has endemic transmission. Reporting rates are calculated by dividing the number of villages with endemic dracunculiasis that report each month by the total number of villages with endemic disease. All villages with endemic dracunculiasis are kept under active surveillance, with daily searches of households for persons with signs and symptoms suggestive of dracunculiasis. These searches are conducted to ensure that detection occurs within 24 hours of worm emergence so that patient management can begin to prevent contamination of water. Villages where endemic transmission of dracunculiasis is interrupted (i.e., zero cases reported for ≥12 consecutive months) also are kept under active surveillance for 3 consecutive years.

WHO certifies a country free from dracunculiasis after that country maintains adequate nationwide surveillance for at least 3 consecutive years and demonstrates that no cases of indigenous dracunculiasis occurred during that period. As of the end of 2011, WHO had certified 192 countries and territories as free from dracunculiasis (3); 14 countries remain to be certified.

Substantial progress has been made since 1986 in reducing the annual number of reported dracunculiasis cases. The 1991 and 2004 WHA goals to eradicate dracunculiasis globally by 1995 and 2009, respectively, were not achieved (6,7). Nevertheless, considerable progress toward eradication continues to be made. The number of cases of dracunculiasis worldwide reported by countries in which the disease is endemic decreased 49%, from 1,058 cases in 2011 to 542 cases in 2012. In January–June 2013, the 89 cases reported from 28 villages in the four remaining dracunculiasis-endemic countries (Chad, Ethiopia, Mali, and South Sudan) represent reductions of 77% and 45%, respectively, from the 393 cases reported from 51 villages during January–June 2012. Of the 89 cases reported during January–June 2013, 83% were from South Sudan.

Chad was officially declared dracunculiasis-endemic again in 2012 as a result of having an indigenous case† for the third consecutive year following discovery of cases in 2010. Chad, Ethiopia, and Mali have each reported slightly more cases in January–June 2013 than in the same period of 2012. Active surveillance for dracunculiasis conducted by the national eradication program in Mali deteriorated significantly after a coup d’etat in March 2012. Active surveillance in at-risk areas of Chad improved dramatically during the same period, and active surveillance in Ethiopia remained weak outside of one known dracunculiasis-endemic district. CDC has tested 92 specimens from suspected cases in nine countries during January 2012–June 2013, of which 50 were determined to be *D. medinensis*.

## Country Reports

### South Sudan

The 10 southern states of the former Sudan became the independent Republic of South Sudan on July 9, 2011. The area of South Sudan reported all of the indigenous dracunculiasis cases notified from the former Sudan since 2002. The South Sudan Guinea Worm Eradication Program (SSGWEP) reported 521 cases in 2012, of which 336 (64%) were contained (Table 1), which was a reduction of 49% from the 1,028 cases reported in 2011. For January–June 2013, SSGWEP reported a provisional total of 74 cases (70% contained) from 52 villages, compared with 389 cases (66% contained) reported from 215 villages in January–June 2012; a reduction of 81% in cases and 76% in the number of villages reporting cases (Table 2). South Sudan reported its first month with zero cases of dracunculiasis in January 2013. Of all cases reported in the first 6 months of 2013, 81% were from only one county, Kapoeta East County, in Eastern Equatoria state.

The peak transmission season in South Sudan now is March–July. In May 2012, the collapse of a key bridge on the only available road for transporting SSGWEP supplies and materials and humanitarian aid to communities in the eastern end of Kapoeta East County added a new challenge to efforts to eradicate dracunculiasis in South Sudan. SSGWEP also faces ongoing challenges in the seasonal movements of persons among villages, gardens, farms, bull cattle camps, milk-cow cattle camps, and grazing areas for smaller livestock such as goats, plus unpredictable population displacements from interethnic cattle rustling raids. The program has continued to intensify interventions (e.g., temephos was used in 85% of dracunculiasis-endemic villages in 2011 and 96% in 2012) and supervision (e.g., 68 national program officers and technical assistants in 2011 and 98 in 2012) as the number of villages in which dracunculiasis is endemic continues to shrink. Unlike the four other currently dracunculiasis-endemic countries, South Sudan does not yet offer a cash reward for reporting a case of dracunculiasis.

### Mali

Mali’s Guinea Worm Eradication Program reported four indigenous cases in 2012, which, in addition to three cases reported by Niger in September 2012 that were exported from Mali, represent a reduction of 42% from the 12 indigenous cases reported in 2011. All three of the exported cases reported in Niger were contained; three of the four cases reported in Mali were contained. Mali reported four cases in January–June 2013, of which only one was contained, compared with one case (contained) reported during January–June 2012. One of the cases (not contained) reported in 2013 was from Mopti Region, and three cases were from Kidal Region.

Mali’s peak transmission season is June–October. The program has not been fully operational in three dracunculiasis-endemic northern regions (Gao, Kidal, and Timbuktu) since April 2012, following a coup d’etat. Periodic humanitarian missions by the United Nations have allowed limited surveillance in areas around the town of Kidal, and parts of Gao and Timbuktu regions recently have become accessible to the program. The most recent sampling of knowledge about the cash reward for reporting a case of dracunculiasis found 70%–90% awareness in areas in which dracunculiasis is endemic (2012) and 0%–2% awareness in areas in which it is not endemic (2011).

### Ethiopia

Ethiopia reported four cases (two contained) in April, May, August, and December 2012, after 9 consecutive months with no known cases. This was a reduction of 33% from the six indigenous cases reported in 2011. The program reported six cases (50% contained) during January–June 2013, compared with two cases reported during the same period of 2012. Five of the six cases in 2013 involved residents of a hamlet where a worm emergence was associated with an uncontained case in April 2012. The sixth case involved a resident of a village that had not reported a case since 2010.

The peak transmission season in Ethiopia is March–May. The only known dracunculiasis-endemic village in 2012 received a functioning borehole well in May 2013. After discussions during the World Health Assembly in May 2013, follow-up visits to Gambella by the federal minister of health, and a visit by a delegation of representatives from The Carter Center, WHO, and the Bill & Melinda Gates Foundation, the health ministry plans to designate staff devoted full time to eradication of dracunculiasis. The most recent available sampling of reward awareness found 83% awareness in an area in which dracunculiasis is endemic (2011) and 60% awareness in an area in which it is not endemic (2012).

What is already known on this topic?

The number of new cases of dracunculiasis (Guinea worm disease) occurring worldwide each year has decreased from an estimated 3.5 million in 1986, when the World Health Assembly declared global elimination as a goal, to 542 in 2012.

What is added by this report?

The number of dracunculiasis cases reported worldwide in 2012 declined by 49%, compared with 2011, and by 77% from January–June 2012 to January–June 2013. Transmission remains endemic in four countries, with South Sudan accounting for 83% of all reported cases during January–June 2013.

What are the implications for public health practice?

Although earlier target dates for global dracunculiasis eradication were missed, progress is accelerating, and eradication is likely within the next few years if disruption of program operations can be minimized, particularly in northern Mali.

### Chad

Chad was officially declared dracunculiasis-endemic again in 2012 after cases of dracunculiasis were confirmed in 3 consecutive years (2010–2012),§ after a decade with no reported cases (8). Chad reported 10 cases (four contained) in nine villages in 2012, compared with 10 cases (four contained) reported from nine villages in 2011, but only two of the 16 villages had cases in both years. Specimens from several cases were confirmed at CDC as *D. medinensis*. Chad reported five cases in January–June 2013, of which four were contained, from five villages, compared with one case reported during January–June 2012. None of the villages reporting cases in 2013 had reported a case previously.

The peak transmission season in Chad appears to be April–August. Since March 2012, The Carter Center has helped the ministry of health to implement active village-based surveillance by training nearly 2,000 volunteers in 700 villages in the at-risk area along the Chari River. In addition to the unusually sporadic, limited nature of the outbreak in Chad over the past 3½ years, dogs with emerging worms have been detected in the same at-risk area in the past year, often without any correlation with villages where human cases have occurred. The worms emerging from dogs are morphologically and genetically indistinguishable from the Guinea worms emerging from humans. Intensive epidemiologic investigation and further genetic studies of these worms are being conducted. The most recent sampling of reward awareness found 100% awareness in an area in which dracunculiasis is endemic (2012) and 38% awareness in an area in which it is not endemic (2012).

### Editorial Note

Based on the trend for 2012, when approximately three quarters of all reported cases occurred during January–June, and initial findings for the same period of 2013, fewer than 150 cases of dracunculiasis likely will be reported in 2013. If so, this would be a historic low. The rapid acceleration in reduction of cases in South Sudan, despite many challenges, is encouraging, and shows that the intensification of interventions there in 2012 is having positive results. Unless Chad, Ethiopia, and Mali can overcome their own challenges quickly, South Sudan might eliminate dracunculiasis before they do.

The main challenges requiring urgent attention by governments and partners include 1) failures in surveillance and containment (e.g., missed cases, unexplained sources of cases, and uncontained cases), 2) establishment and maintenance of surveillance in Guinea worm–free areas of all countries in which the disease still occurs or was recently eliminated, and 3) providing clean drinking water quickly to as many targeted villages as possible. Insecurity in parts of Mali is now the main political barrier to complete eradication of dracunculiasis.

## Figures and Tables

**TABLE 1:** Number of reported dracunculiasis cases, by country and local interventions — worldwide, 2012

Country	Reported cases			Change in indigenous cases in villages/localities under surveillance during the same period in 2011 and 2012 (%)	Villages under active surveillance in 2012					Status of interventions in endemic villages in 2012					
		
	Indigenous in 2012	Imported in 2012*	Contained during 2012 (%)		No.	Reporting monthly (%)	Reporting ≥1 cases	Reporting only imported cases†	Reporting indigenous cases	Endemic villages 2011–2012	Reporting monthly§ (%)	Filters in all households§ (%)	Using temephos§ (%)	≥1 sources of safe water§ (%)	Provided health education§ (%)
South Sudan	521	0	(64)	(−49)	6,410	(100)	255	166	89	167	(100)	(100)	(96)	(33)	(98)
Mali¶	7	0	(86)	(−42)	121	(88)	3	0	3	9	(78)	(78)	(57)	(71)	(78)
Chad	10	0	(40)	(0)	693	(95)	9	0	9	2	(100)	(100)	(100)	(100)	(100)
Ethiopia	4	0	(50)	(−50)	77	(100)	4	2	2	3	(100)	(100)	(100)	(67)	(100)
**Total**	**542**	**0**	**(73)**	**(−49)**	**7,301**	**(99)**	**271**	**340**	**103**	**181**	**(98)**	**(98)**	**(94)**	**(35)**	**(98)**

* Imported from another country.

† Imported from another country or from another in-country disease-endemic village.

§ The denominator is the number of villages/localities where the program applied interventions during 2011–2012.

¶ In 2012, seven cases were attributed to Mali: four indigenous cases reported by Mali’s Guinea Worm Eradication Program (GWEP) plus three cases reported by Niger in September 2012 that were exported from Mali. GWEP operations (supervision, surveillance, and interventions) were interrupted in Mali’s Kidal, Gao, and Timbuktu regions as a result of a coup d’etat, beginning in April 2012.

**TABLE 2:** Number of reported indigenous dracunculiasis* cases, by country — worldwide, January 2011–June 2013

Country	2011	2012*	1-yr change (%)	January–June 2012*	January–June 2013	6-mos change (%)	Cases contained during January–June 2013 (%)
South Sudan	1,028	521	(−49)	389	74	(−81)	(70)
Mali†	12	7	(−42)	1	4	(300)	(25)
Chad	10	10	(0)	1	5	(400)	(80)
Ethiopia	6	4	(−33)	2	6	(200)	(50)
**Total**	**1,056**	**542**	**(−49)**	**393**	**89**	**(−77)**	**(67)**

* In 2012, three cases were imported into Niger from Mali and are included in Mali’s total. These persons were residents in Mali the preceding year, and Niger interrupted transmission of Guinea worm disease in 2008. No reports of cases imported from one country to another were reported during January–June 2013.

† Guinea Worm Eradication Program operations (supervision, surveillance, and interventions) were interrupted in Kidal, Gao, and Timbuktu regions as a result of a coup d’etat, beginning in April 2012.

## Footnotes

* Transmission from a patient with dracunculiasis is contained if all of the following conditions are met: 1) the disease is detected <24 hours after worm emergence; 2) the patient has not entered any water source since the worm emerged; 3) a volunteer has managed the patient properly, by cleaning and bandaging the lesion until the worm has been fully removed manually and by providing health education to discourage the patient from contaminating any water source (if two or more emerging worms are present, transmission is not contained until the last worm is removed); and 4) the containment process, including verification of dracunculiasis, is validated by a supervisor within 7 days of emergence of the worm. All of these criteria must be achieved for each emerged worm for the case to be considered contained.

† An indigenous case is defined as infection occurring in a person exhibiting a skin lesion or lesions with emergence of one or more Guinea worms in a person who had no history of travel outside his or her residential locality during the preceding year.

§ A country will be considered to have reestablished dracunculiasis endemicity if 1) the country has not reported a confirmed indigenous case of the disease for >3 years, and 2) subsequent indigenous transmission of cases (laboratory-confirmed) is shown to occur in that country for ≥3 consecutive calendar years.
